# Effects of Non-Sport-Specific Versus Sport-Specific Training on Physical Performance and Perceptual Response in Young Football Players

**DOI:** 10.3390/ijerph18041962

**Published:** 2021-02-18

**Authors:** Damiano Formenti, Alessio Rossi, Tindaro Bongiovanni, Francesco Campa, Luca Cavaggioni, Giampietro Alberti, Stefano Longo, Athos Trecroci

**Affiliations:** 1 Department of Biotechnology and Life Sciences, University of Insubria, 21100 Varese, Italy; damiano.formenti@uninsubria.it; 2 Department of Computer Science, University of Pisa, 56126 Pisa, Italy; alessio.rossi2@gmail.com; 3 Department of Biomedical Sciences for Health, Università Degli Studi di Milano, 20129 Milano, Italy; tindaro.bongiovanni@gmail.com (T.B.); cavaggioni.luca@gmail.com (L.C.); giampietro.alberti@unimi.it (G.A.); stefano.longo@unimi.it (S.L.); athos.trecroci@unimi.it (A.T.); 4 Department for Life Quality Studies, University of Bologna, 47921 Rimini, Italy; 5 IRCCS Istituto Auxologico Italiano, Obesity Unit and Laboratory of Nutrition and Obesity Research, Department of Endocrine and Metabolic Diseases, 20149 Milano, Italy

**Keywords:** motivation, youth training, physical activity, enjoyment

## Abstract

This study aimed to compare the effects of non-sport-specific and sport-specific training methods on physical performance and perceptual response in young football players. Seventy-nine under 11 participants were selected and assigned to non-sport-specific (NSSG), sport-specific (SSG), and control (CNTG) groups. The NSSG training protocol consisted of combined stimuli based on balance, agility, and jump rope drills. The SSG training protocol included technical exercises, defensive and offensive game-based drills, and a small-sided game. The CNTG included the participants not taking part in any sport training. All participants were tested for general motor coordination (Harre test), dynamic balance (Lower Quarter Y-balance test), and dribbling before and after 10 weeks of training (NSSG and SSG) or habitual activity (CNTG). At post-intervention, perceived enjoyment was requested by the Physical Activity Enjoyment Scale (PACES). A two-way repeated measure analysis of covariance was used to detect interactions and main effects of time and groups controlling for baseline values. Whereas, a one-way analysis of variance was used to evaluate PACES-related differences between groups. NSSG gained greater improvements (*p* < 0.05) compared with SSG in the Harre and Lower Quarter Y-balance tests, while dribbling skills improved similarly in both groups. Regarding PACES, NSSG and SSG presented a comparable perceived enjoyment. These findings suggest that a 10-week non-sport-specific training is an enjoyable practice capable to promote greater improvements in general motor coordination and dynamic balance compared with sport-specific training in youth football players. This can occur without impairment of football-specific skills.

## 1. Introduction

Youth football players perform several specific-to-general activities (e.g., shooting, dribbling, jumping, and changing direction) requiring a certain technical proficiency [1] and physical readiness to cope with the demands of the game [2]. According to this, youth training is important to provide young players, especially those preadolescents, with a balanced development of both general and sport-specific stimuli, distinctly during the sampling years (from 6 to 12 years) of the youth developmental model of sports participation [3,4]. It has been recognized that young individuals experiencing comprehensive physical activities during their development stages required less time to reach a sport-related motor proficiency [5]. In this context, concurrent all-embracing activities based on non-sport-specific stimuli (e.g., exercises without using the ball in football) may help to better improve physical fitness and physiological conditions in individuals regularly practicing a specific sport [5,6]. It was previously found that a 12-week period of non-sport-specific speed, agility, and quickness (SAQ) drills significantly improved 5-m sprint and agility compared with sport-specific-only drills in under-11 football players [7]. Moreover, the authors found that SAQ provided comparable levels of perceived enjoyment as football-specific drills [7]. In accordance, developing general motor competence in young football players should involve a combination of closed (e.g., sprinting and change of direction) and open (e.g., balance and reactive movements) drills to offer an appropriate mixture of stimuli (i.e., multidimensional approach) that suits the goals over the sampling years [3]. As suggested, these drills should not be exclusively focused on football-specific stimuli (technical and tactical drills); rather, these drills should be aided also by non-sport-specific stimuli (e.g., without using the ball) taking into account the players’ perceptual response (i.e., perceived enjoyment).

The chance to experience enjoyment through a variety of physical activities (mainly within an all-embracing non-sport-specific approach) plays a key role in increasing youth’s sports engagement [8,9] in both recreational and competitive environments. Additionally, it has been observed enjoyment as pivotal to sport commitment [10]. Indeed, according to Michael et al. [8], enjoyment could mediate the link between team sport-like disciplines probably due to its influence also on the social interactions [8]. In this wake, youth team sports training should also contribute to induce positive perceptual responses in terms of enjoyment, thus turning the young athletes’ sport participation into a long-term commitment [11]. This condition passes through the use of principle and practice based on the enjoyment to motivate youth [11,12]. Tjomsland et al. [12] considered enjoyment as the central part of the overall sports experience being an expectation that prompts young players to start and continue engaging with football-specific activities. However, despite the importance of such enjoyment responses together with the need for all-embracing activities (non-sport-specific training) for physical and perceptual well-being, the information in the literature is scarce and demands further insights. Additionally, from a practical point of view, it appears that coaching practice often promotes technical and tactical teaching at the expense of complete and harmonious development of physical abilities with limited attention to perceptual responses [13].

In this context, this study aims to compare the effects of non-sport-specific with sport-specific training on physical performance and enjoyment in young football players. We hypothesized that non-sport-specific practice would be superior in improving physical performance exhibiting a comparable perception of enjoyment as football-specific training.

## 2. Materials and Methods

### 2.1. Subjects

Seventy-nine healthy male participants were selected. Then, the participants were randomly assigned to a non-sport-specific group (NSSG, *n* = 27; age = 10.6 ± 0.35 years, body mass = 41.6 ± 5.0 kg, height = 1.43 ± 0.05 m) to a sport-specific group (SSG, *n* = 26; age = 10.5 ± 0.40 years, body mass = 42.0 ± 5.6 kg, height = 1.43 ± 0.04 m), and to a control group (CNTG, *n* = 26; age = 10.3 ± 0.50 years, body mass = 39.6 ± 6.0 kg, height = 1.42 ± 0.06 m). The participants belonging to NSSG and SSG were football players regularly engaged in a football training routine (at least 6 h per week) with a minimum of 4 years of training background. CNTG participants were not engaged in sports activities. Unfortunately, two individuals (1 of SSG and 1 of CNTG) did not complete the study for external reasons. Thereby, the individual sample size of SSG and CNTG came down to 25 participants. All individuals and their parents were informed about the purposes and the experimental procedures of the study. Parents or legal guardians signed and provided the written informed consent before starting the experimental investigation. The Ethics Committee of the local University approved (approval number 32/16) the study in compliance with the Helsinki Declaration.

### 2.2. Procedures

**Figure 1:**
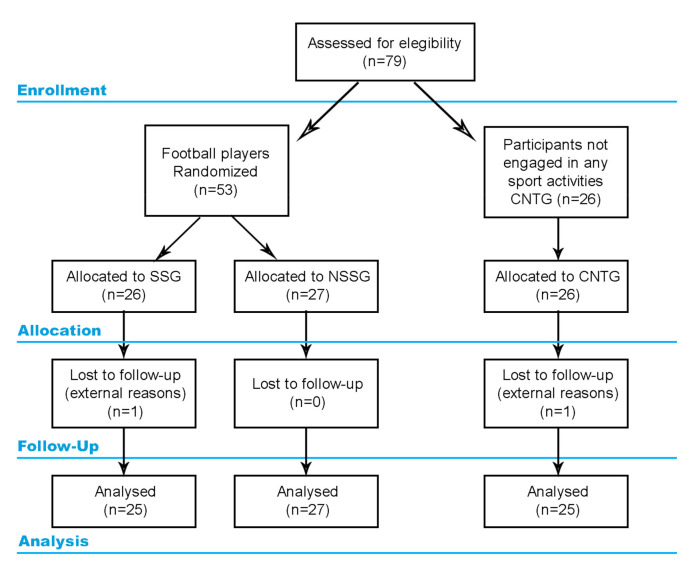
Graphical representation of the study design. NSSG = non-sport-specific group, SSG = sport-specific group.

In this study, a pre-to-post parallel quasi-randomized design was adopted to compare the effects of different training interventions (non-sport-specific versus sport-specific) on physical performance and perceptual response. The study design is represented in Figure 1. A 4-week familiarization period was administered before the training intervention, in which the participants got accustomed to the experimental protocol [7]. Then, a testing day was planned to include general motor coordination (Harre circuit test), dynamic balance (Lower Quarter Y-Balance test), and technical skills (dribbling test) evaluations [14,15] in a random order. Prior to testing, each participant warmed up for 5 min by FIFA 11+-related running drills to get accustomed to the subsequent effort [2]. A 10-min rest period was given between each physical performance assessment. The perceptual enjoyment based on the enjoyment rating was obtained after the experimental period.

### 2.3. Experimental Protocol

The experimental protocol consisted of 2 interventions per week over 10 weeks (20 sessions in total lasting ~30 min each) from February to June during the competitive season. At the time of the experimental period, the regular competitive week included 3 training sessions (lasting 90 min each) and a match-play per week for NSSG and SSG. The training interventions underwent by NSSG and SSG were administered at the beginning of the first 2 weekly training sessions (e.g., on Monday and Wednesday) after a brief warm-up [16]. Then, all participants continued their regular training based on football-related drills, consisting of technical activity (i.e., dribbling, passing, and shooting drills) and game formats (i.e., small-sided games), which were identical for both groups. According to the footballer’s training routine, they underwent a third weekly training session (e.g., on Friday) including the same football-specific contents [17]. This setting allowed us to keep the ecological validity of the study high [7,16,17]. Each training session occurred at the same time of day (4.30 p.m. to 6.00 p.m.).

Non-sport-specific training. In the NSSG, the training protocol was a combination of training elements based on balance, SAQ, and jump rope drills (Table 1) lasting ~30 min. The training duration of these elements was ~8 min each with a 2 min of rest in between.

Balance drills were arranged with a work:rest ratio of 1:1 [16], in which the participants performed overall 8 × 30 s of work followed by 30 s of rest. The training volume was kept constant throughout the weeks, while the exercise execution changed (from balancing to slacklining) to increase their complexity (Table 1). SAQ drills consisted of brief efforts (from 3 s to 5 s) in the form of speed, agility, and quickness drills (Table 1) arranged with a work:rest ratio of 1:2 during a single repetition [7]. The number of drills, training volume, and rest periods were similar to the framework previously adopted [7]. Training volume and exercise intensity increased throughout the weeks by manipulating the number of change of directions and sprint distance from non-cognitive to cognitive demanding contexts [7] (Table 1). The jumping rope drills consisted of 5 exercises (2 per session) performed within the 10-week period (Table 1) using the same rope (mass = 230 g; material, Polyvinyl Chloride) [14]. All exercises were arranged with a work:rest ratio of 1:1 in which the participants performed 4 reps × 30 s of work followed by 30 s of rest for each exercise [14]. The training volume was kept constant throughout the weeks, while the exercise execution changed to increase their complexity (Table 1). A certified strength and conditioning coach administered the entire experimental protocol to ensure a proper exercise execution providing the subjects with technical support and verbal encouragement.

*Sport-specific training.* In the SSG, the football-specific training was divided into three parts and included technical exercises, a combination of defensive and offensive game-based drills (with and without a goalkeeper), and a small-sided game. Each element of the program lasted ~8 min interspersed by 2 min of rest in between. The technical exercises involved ball mastery (from week 1 to week 2), passing and receiving drills (from week 3 to week 4), ball carrying and dribbling drills (from week 5 to week 6), dribbling and feinting drills (from week 7 to week 8), and crossing and shooting drills (from week 9 to week 10). The game-based drills were arranged with and without goalkeeper by increasing the number of players (from 1 versus 1 to 3 versus 3) throughout the 10-week period (Table 2). Then, a 1 × 8-min 4-a-side small-sided game was administered as a regular match (with two goalkeepers and free play) (Table 2). Where not possible to adhere to the 4-a-side format one played as a wildcard due to the number of participants.

#### 2.3.1. Physical Performance Assessment

The choice of the present physical performance tests (Harre circuit test, Lower Quarter Y-balance test, and linear dribbling test) was based on their easiness, reliability [14,18,19], and enjoyability. Moreover, they can provide practical information on general motor coordination, dynamic balance, and dribbling skills.

The Harre circuit test was used to assess the ability of an individual to organize and coordinate complex movements (e.g., somersaulting, climbing, hopping, landing, running, and turning) with a high number of joints involved and levels of force generated [14,20]. After an initial somersault, participants performed 3 consecutive passages above and below 3 obstacles by turning around a central cone, then sprinted back to the starting line. Three trials separated by 5 min of rest were executed. The total time of each trial was recorded by using a timing gate system (Witty system, Microgate, Bolzano, Italia). The best performance time was considered in the analysis. In case of mistakes (e.g., touching the obstacle), the participants were asked to repeat the trial after 2 min of rest. All trials were performed in an indoor gym, observing the same conditions [14].

The lower Quarter Y-balance test (YBT-LQ) was employed to assess dynamic balance [21,22]. The distance reached in anterior (A), posteromedial (PM), and posterolateral (PL) directions were measured for each leg. Participants performed the YBT-LQ barefoot with their hands placed on the hips to avoid influences of footwear on balancing. Three trials standing on the right and left foot in all directions were performed. Individuals were instructed not to touch the floor with the reach foot except for marking the actual reach distance. In case of a failure, the trial was repeated after 2 min of rest. A composite score was obtained by the following formula: composite score = [(A + PM + PL)/3 × leg length] × 100 [18]. The average score was considered for the analysis.

#### 2.3.2. Technical Skills

A linear dribbling test was employed to assess participants’ ability to rapidly dribble the ball [19]. The participants dribbled the ball down a corridor 1.5 m wide and 20 m long across two cones. Then, after passing the cones, the individual had to stop the ball within 2 m after the finish line to complete the test. This served to avoid sprinting without keeping the ball near the leading foot upon arrival. In case of temporary exit of the ball from the boundaries, the trial was invalidated and done again after 2 min of rest. An electronic timing gate system positioned at the starting and finish lines recorded each dribbling time. Three trials were executed, and the best performance was considered in the analysis.

### 2.4. Perceptual Response

The Italian version of the Physical Activity Enjoyment Scale (PACES) was administered to detect the participants’ perceived enjoyment after the training intervention. The PACES consisted of 16 items (9 positive and 7 negative). For instance, the positive items referred to statements like “I enjoy it”, “I find it pleasurable”, “It gives me energy”, “It’s very pleasant”, “My body feels good”, “I get something out of it”, “It’s very exciting”, “It gives me a strong feeling of success”, and “It feels good”. These items were scored on a 5-point Likert scale ranging from 1 (“Disagree a lot”) to 5 (“Agree a lot”) and were related to the same stem: “When I am active… [name of the training intervention for NSSG and SSG or the activity engaged in the free time for CNTG]…” [23,24]. The PACES score was obtained by the ratings of the positive items [16].

### 2.5. Statistical Analysis

Data normal distribution was verified by the Shapiro–Wilk test. Absolute reliability was performed using the coefficient of variation (CV) calculated as [standard deviation/mean) × 100]. A one-way ANOVA was used to detect differences in the baseline physical performance values between groups. In case of difference, a two-way repeated measure on one factor (time) analysis of covariance (ANCOVA RM) was used to detect interactions (time x intervention) and main effects of time (within-group changes) and groups (NSSG, SSG, and CNTG), controlling for baseline values (covariance factor). A one-way ANOVA was then used to evaluate potential group differences in the PACES score. Bonferroni post-hoc tests were used for multiple comparison purposes. Significance was set with *p* ≤ 0.05. The analysis was performed using the GraphPad Prism 7.0 software (GraphPad Software, Inc., San Diego, CA, USA). The Hedge’s g effect size [25] between each performance change was calculated, and threshold values were g < 0.2 (trivial), 0.2 < g < 0.5 (small), 0.5 < g < 0.8 (moderate), and g > 0.8 (large). Data are presented as mean ± SD. Relative changes were expressed as means ± 95% confidence intervals (CI).

## 3. Results

**Figure 2:**
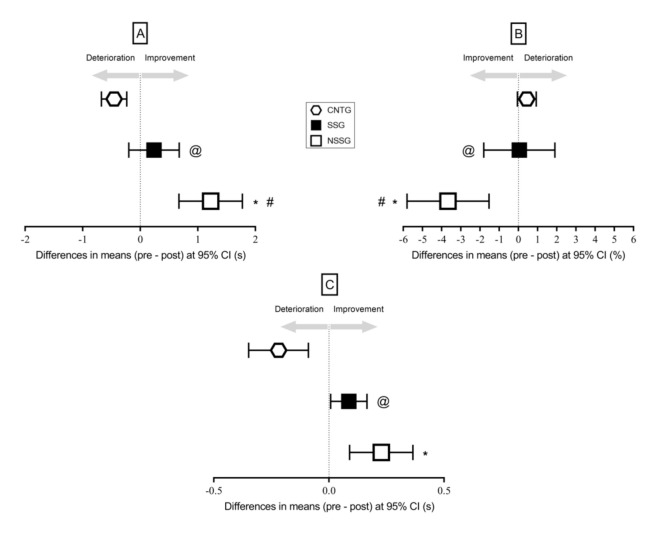
Graphical explanation of the pre-to-post difference with 95% confidence intervals (95% CI) for Harre test (panel (**A**)), YBT-LQ (panel (**B**)), and dribbling test (panel (**C**)). * Significant (*p* < 0.001) difference between NSSG and CNTG from post-hoc analysis. # Significant (*p* < 0.05) difference between NSSG and SSG from post-hoc analysis. @ Significant (*p* < 0.05) difference between SSG and CNTG from post-hoc analysis. Note: YBT-LQ = Lower Quarter Y-balance test, NSSG = non-sport-specific group, SSG = sport-specific group, CNTG = control group.

The CVs were 3.2% (CI from 1.9% to 4.5%), 2.6% (CI from 1.8% to 3.4%), 3.1% (CI from 1.9% to 4.3%) for the Harre test, YBT-LQ, and dribbling test, respectively. All descriptive statistics about pre-to-post testing outcomes are shown in Table 3. The ANOVA RM revealed a significant (F = 6.07, *p* < 0.001) interaction in the running time to complete the Harre test. Bonferroni post-hoc analysis (Figure 2) showed that NSSG presented lower running time compared to SSG (*p* = 0.005, g = 1.42) and CNTG (*p* < 0.001, g = 2.56, large). SSG also presented significant lower time compared to CNTG (*p* = 0.004, g = 2.72) (Figure 2, large). Regarding YBT, significant interaction was found (F = 6.11, *p* = 0.004). Post-hoc analysis showed that NSSG presented a higher composite score compared to SSG (*p* = 0.027, g = 1.93, large) and CNTG (*p* < 0.001, g = 2.20, large). SSG also presented significant higher score compared to CNTG (*p* = 0.024, g = 1.98, large) (Figure 2). A significant interaction was also found in the dribbling test (F = 11.18, *p* < 0.001). Post-hoc analysis showed that both NSSG and SSG dribbled faster than CNTG only (*p* < 0.001, g = 1.22, large, and *p* = 0.001, g = 2.85, large, respectively) (Figure 2). The pre-to-post differences with 95% CI for each test are shown in Figure 2. The one-way ANOVA revealed a significant (F = 36.6, *p* < 0.001) difference between groups in the PACES score. Post-hoc analysis showed that both NSSG and SSG exhibited higher (*p* < 0.001, g = 1.74, large, and g = 1.86, large) scores than CNTG. No significant difference was observed between NSSG and SSG (*p* > 0.05, g = 0.21, small).

## 4. Discussion

The present study sought to investigate the effects of a 10-week period of training with non-sport-specific stimuli compared with sport-specific stimuli on physical performance and perceptual response in young football players. The observed main findings revealed that NSSG performed significantly better than SSG in the Harre and YBT-LQ tests after the 10-week training period. Interestingly, NSSG and SSG did not differ significantly in the dribbling test. Likewise, the PACES score was not significantly different between NSSG and SSG at the end of the experimental period. Overall, the outcomes of the physical performance assessment show that the employment of a 10-week non-sport-specific training period can promote greater improvements in general motor coordination and dynamic balance performance compared with sport-specific training in young football players. This can take place without the football-specific skills (i.e., dribbling) being impaired. From these perspectives, our hypothesis that non-sport-specific practice would be superior in improving physical performance as well as to provide comparable enjoyment perception toward SSG was verified.

In this study, the Harre test was employed to provide information on general motor coordination linked to physical (somersaulting, climbing, hopping, landing, running, and turning) and cognitive (reaction and spatial-temporal awareness) stimuli [14,20]. According to our findings, NSSG performed better than SSG. This result can be compared to those of an earlier study that observed the superiority of a 12-week period of SAQ training in improving physical (i.e., 5 m sprint) and cognitive (i.e., Y-shaped reactive agility test) performance as against a football-specific training in young players [7]. Of note, a recent study investigated the effects of a 12-week game-based training versus multilateral training on technical performance (shuttle dribble test) and physical fitness (standing long jump, shuttle run, and 6-min Mini-Cooper tests) in young athletes playing football [13]. Overall, the authors found no differences between both training modalities both improving dribbling time and jumping and running distances. A related idea that might explain these results may refer to the content of the multilateral training against that employed in the present study by NSSG. Although aimed to develop body perception and physical abilities with non-sport-specific stimuli, the multilateral drills included only physical-related activities such as sprints, relays, and jumps over hurdles. Conversely, the current players taking part in the NSSG underwent a combination of various physical (speed, quickness, and jump rope) and cognitive (balance and agility) drills. Moreover, another explanation might be related to the type of the selected tests, which might be less successful at detecting the changes in physical performance compared with those of our study. Based on a sport pedagogical perspective, young players experiencing training sessions under non-specific approaches have the chance to try several sport disciplines helping themselves to select the preferred sport [26]. This aspect would favor the maintenance of the enjoyment response at high levels. Additionally, based on a performance perspective, Harrison et al. [27] found preadolescents benefited from non-sport-specific stimuli by reaching higher intensities than sport-specific stimuli during a game format. The authors concluded that these stimuli may be considered a useful training tool by coaches to also maximize players’ decision-making abilities [27]. Of note, the use of a wide range of non-sport-specific stimuli has the advantage to expose young players to plenty of physical, cognitive, and psycho-social environments [3,28], in which an individual’s coordinative trait (e.g., motor and postural control, static and dynamic balance, spatial-temporal organization, and body perception) may be broadly developed [20]. Accordingly, the current results on dynamic balance also provide evidence for the latter claim. Training content targeting balance stimuli can help young athletes to increase their body perception and postural control on a more individual level [16]. Our results demonstrated how non-sport-specific training based on various physical and cognitive stimuli (without using the ball) can promote dynamic balance skills to a greater extent than football-specific training in young players.

Another finding of this study is that the activity of the NSSG aroused comparable perceived enjoyment in the young players as that induced by the SSG. A similar result was found by Trecroci and colleagues [16] who compared the effects of slackline training versus traditional balance and football-specific training on physical performance and perceived enjoyment. Although the aim was beyond that of the current study, the authors found the integration of slackline training (based on non-sport-specific stimuli) to the weekly routine inducing equivalent PACES scores as for football-specific training. Then, it might be stated that the use of non-sport-specific stimuli would represent an alternative to exclusive sport-specific stimuli for furthering even-more appealing training strategies [29] and to promote young players’ engagement in sport [16]. However, there is a dearth of information on the potential effects of non-sport-specific stimuli on perceptual well-being (i.e., enjoyment) in young football players. Future research will have to address the impact of each individual stimulus (physical and cognitive) on youth’s perceptual response in the attempt to promote a better enjoyment perception.

An interesting side finding was that NSSG did not affect players’ technical ability (i.e., dribbling task) over the 10-week period. It should be noted the overall training content of NSSG was employed twice a week within the regular weekly training routine. This means that the players taking part in the NSSG were also exposed to football-specific stimuli over the intervention weeks. Hereby, the general picture emerging from this finding is that integrating non-sport-specific contents along with the weekly training routine can promote superior improvements in physical performance than the football-specific training alone, without impacting young players’ dribbling abilities. Indeed, this is also supported by the literature shedding light on the importance of all-embracing activities (non-sport-specific training) for physical and perceptual well-being.

The current study presents main limitations that should be clearly elucidated. General motor coordination was assessed by the Harre test, which encompasses a time-constraint activity. The fact that an individual had to rapidly execute a combination of different tasks (somersaulting, running, climbing, jumping, landing, and turning), would not allow the practitioners to infer detailed information on the quality of the execution (i.e., motor competence), perhaps linked to a specific coordinative trait (e.g., the ability of coupling up different movements). However, the Harre test presents movement tasks that are inherent to a match-play (e.g., hopping, landing, running, and turning) and requires a certain spatial-temporal awareness making useful its employment to infer young athletes’ general motor coordination in football [14]. Additionally, although the present protocols of NSSG and SSG embraced cognitive stimuli (decision-making demands by agility and small-sided games, respectively), we did not provide any measure of it. Being cognitive functions pivotal in preadolescents, especially in team sport athletes, the inclusion of a specific assessment (e.g., visual search) would have contributed to understanding the potentiality of an all-embracing approach (i.e., non-sport-specific training) within a field-based context.

## 5. Conclusions

The current findings provide evidence that performing 10 weeks of non-sport-specific training can promote greater improvements on general motor coordination and dynamic balance than sport-specific training in youth football players. Additionally, our findings provide theoretical support for the integration of non-sport-specific contents along with the youth weekly football training routine having the advantage to retain a comparable level of technical skills (i.e., dribbling) and perceptual well-being (i.e., enjoyment).

## Figures and Tables

**Table 1:** Training content of the 10-week intervention performed by the NSSG.

Weeks	Balance Drills	SAQ Drills	Jump Rope Drills
Week 1 to 2	One-leg standing on unstable surfaces (i.e., trampoline and wobble board) while moving the upper body with and without eyes open.	Basic footwork exercises (split-steps, line drills, lateral line, and multiple hops) with no equipment followed by brief linear sprints over 5 m	Basic bounce steps Double basic bounce steps
Week 3 to 4	From an unstable surface: (a) short jumps with a 90° body rotation and landing on a single stance; (b) short jumps with a 180° body rotation and landing on single-leg stance	Basic footwork exercises (skipping, hopscotch, in&out drills) over the speed-ladder followed by brief sprints with 1–3 change of directions at 30° and 45° over 10 m	Double basic bounce steps Alternate bounce steps
Week 5 to 6	Stepping forward and backward with and without assistance on the slackline	Advanced footwork exercises (foot exchange, icky shuffle, hip twist) over the speed-ladder followed by brief sprints with 3–5 change of directions at 30°, 45°, and 90° over 10 m	Alternate bounce steps Scissors steps
Week 7 to 8	Walking forward and backward with and without assistance on the slackline	Combination of basic and advanced footwork exercises with basic agility drills in response to stimuli (brief acceleration and deceleration)	Scissors steps Double under steps
Week 9 to 10	Lowe-limb swinging while standing on a single leg stance with and without assistance on the slackline	Combination of basic and advanced footwork exercises with advanced agility drills in response to stimuli (chasing runs and mirror drills)	Double under steps

Note: NSSG = non-sport-specific group, SAQ = speed agility and quickness.

**Table 2:** Training content of the 10-week intervention performed by the SSG.

Weeks	Technical Drills	Game-Based Drills	Small-Sided Game Drills
Week 1 to 2	Ball mastery	1 versus 1 (no goalkeeper)	4 versus 4 Regular match
Week 3 to 4	Passing and receiving	1 versus 1 2 versus 1 (with goalkeeper)	
Week 5 to 6	Ball carrying and dribbling	2 versus 1 2 versus 2 (no goalkeeper)	
Week 7 to 8	Dribbling and feinting	2 versus 2 3 versus 2 (with goalkeeper)	
Week 9 to 10	Crossing and shooting	3 versus 2 3 versus 3 (no goalkeeper)	

Note: SSG = sport-specific group.

**Table 3:** Descriptive statistics of the Harre circuit test, Lower Quarter Y-balance test (YBT-LQ), linear dribbling test, and Physical Activity Enjoyment Scale (PACES) for the non-sport-specific group (NSSG), sport-specific group (SSG), and control group (CNTG).

Groups		Physical Performance Assessment		Technical Skills	Perceptual Response
	Time Points	Harre Circuit Test (s)	YBT-LQ (cm)	Linear Dribbling Test (s)	PACES (u.a.)
NSSG	PRE	17.7 ± 2.32	96.7 ± 4.53	5.32 ± 0.88	-
	POST	16.5 ± 1.62	100.0 ± 5.74	5.09 ± 0.73	42.9 ± 2.40
SSG	PRE	17.3 ± 1.38	99.6 ± 6.34	5.10 ± 0.61	-
	POST	17.0 ± 1.59	99.6 ± 7.40	5.02 ± 0.58	43.4 ± 2.33
CNTG	PRE	21.6 ± 3.78	78.1 ± 6.92	8.91 ± 0.92	-
	POST	22.1 ± 3.91	77.6 ± 6.89	9.13 ± 1.02	35.0 ± 5.94

## Data Availability

The data presented in this study are available on request from the corresponding author.
